# Supplementary material for: A breeding pool of ideas: Analyzing interdisciplinary collaborations at the Complex Systems Summer School
Source: PLoS One. 2021 Feb 1;16(2):e0246260. doi: 10.1371/journal.pone.0246260 (PMC7850475; doi:10.1371/journal.pone.0246260)

# A Group Degree Centrality Null Models for All Attributes

S1 Fig: Comparison of group degree centrality values aggregated by participant discipline to null model. Dots correspond to actual eigencentrality value. Grey error bars represent 95th percentile confidence intervals from the null model.

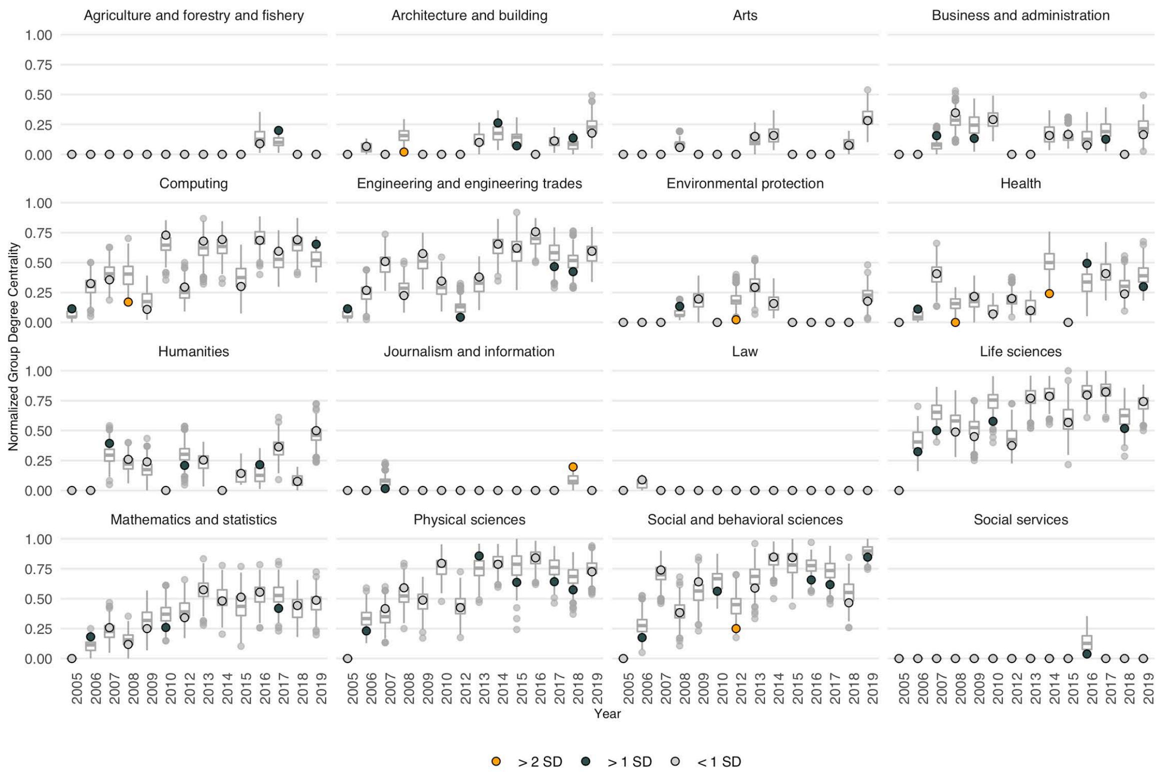

S2 Fig: Comparison of group degree centrality values aggregated by participant gender to null model. Dots correspond to actual eigencentrality value. Grey error bars represent 95th percentile confidence intervals from the null model.

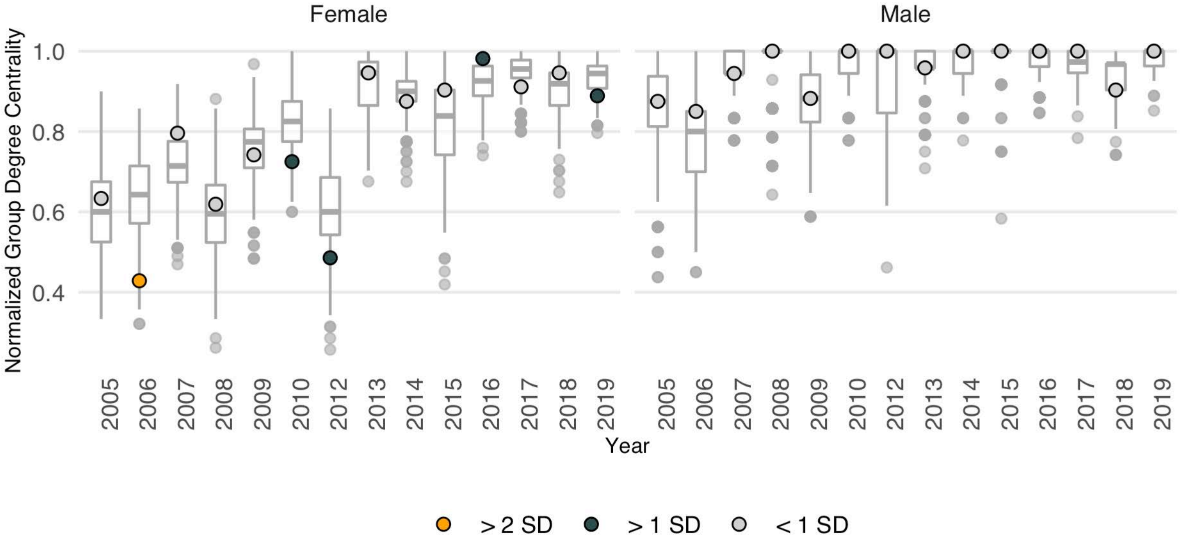

S3 Fig: Comparison of group degree centrality values aggregated by participant position to null model. Dots correspond to actual eigencentrality value. Grey error bars represent 95th percentile confidence intervals from the null model.

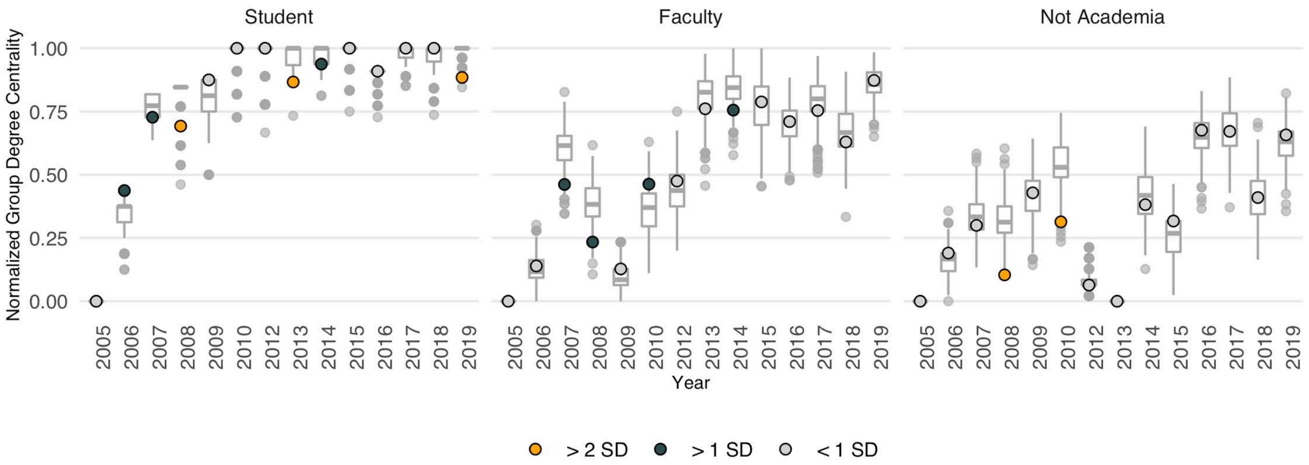

S4 Fig: Comparison of group degree centrality values aggregated by participant institutional prestige to null model. Dots correspond to actual eigencentrality value. Grey error bars represent 95th percentile confidence intervals from the null model.

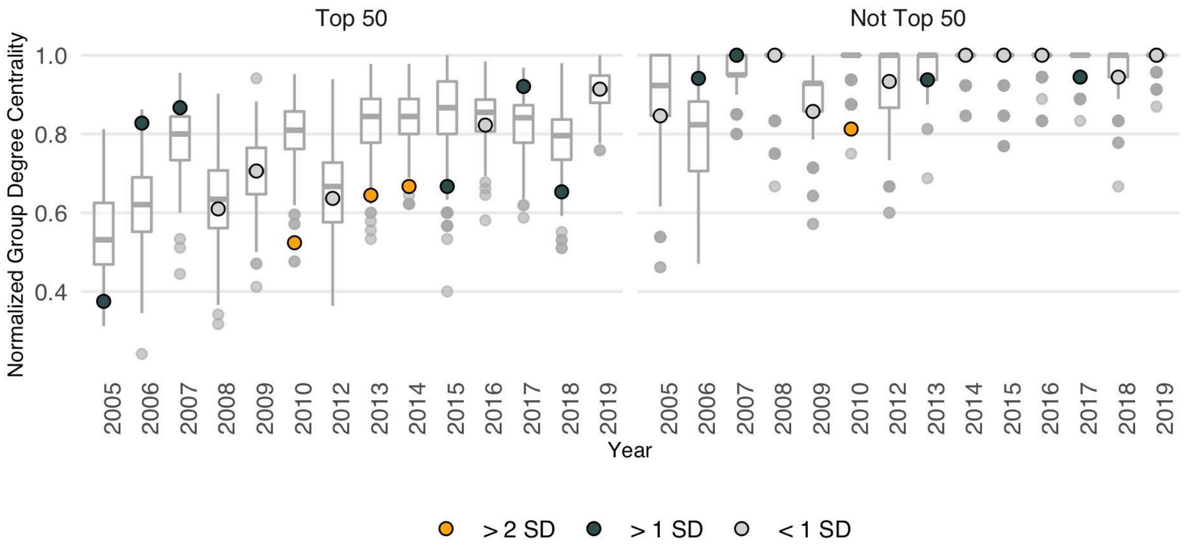

S5 Fig: Comparison of group degree centrality values aggregated by participant country of study. Dots correspond to actual eigencentrality value. Grey error bars represent 95th percentile confidence intervals from the null model.

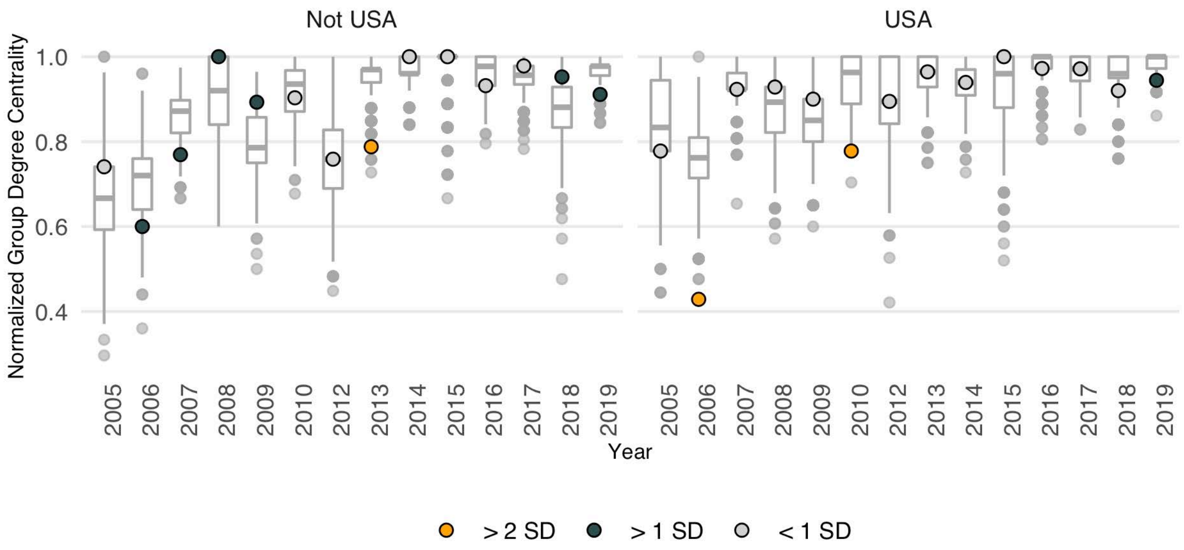

# B Eigencentrality Null Models for All Attributes

S6 Fig: Comparison of eigencentrality values aggregated by participant discipline to null model. Dots correspond to actual eigencentrality value. Grey error bars represent 95th percentile confidence intervals from the null model.

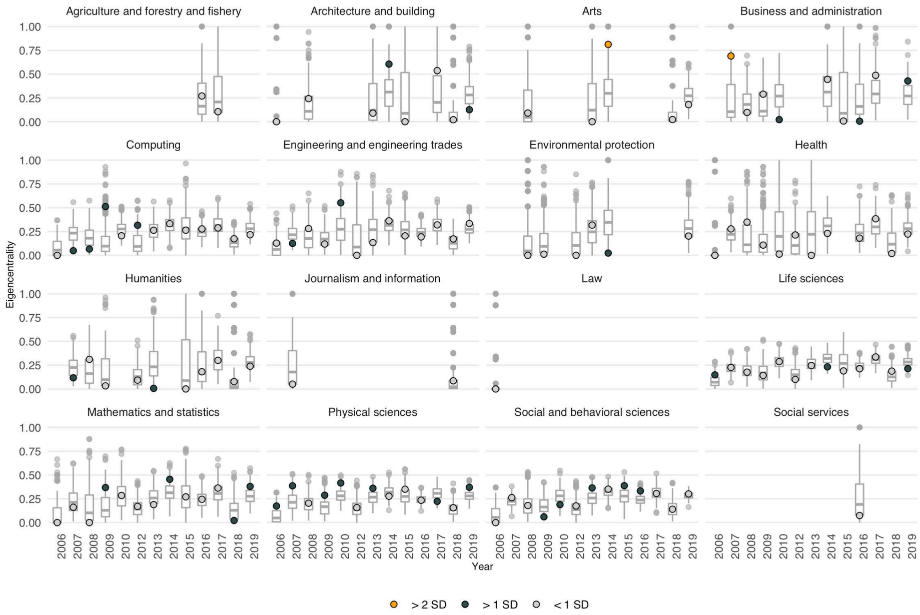

S7 Fig: Comparison of eigencentrality values aggregated by participant gender to null model. Dots correspond to actual eigencentrality value. Grey error bars represent 95th percentile confidence intervals from the null model.

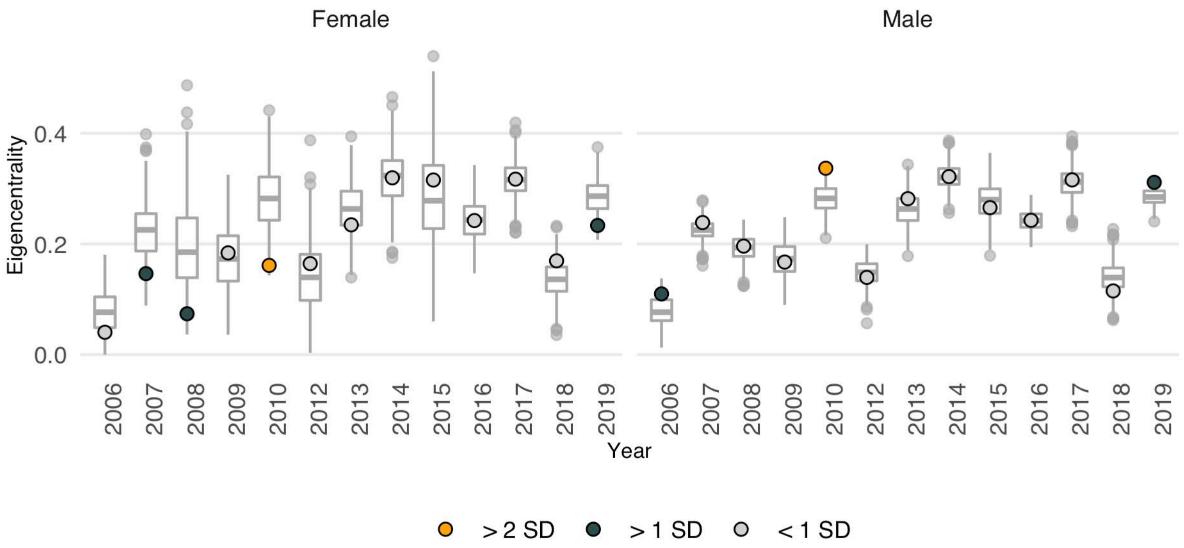

S8 Fig: Comparison of eigencentrality values aggregated by participant position to null model. Dots correspond to actual eigencentrality value. Grey error bars represent 95th percentile confidence intervals from the null model.

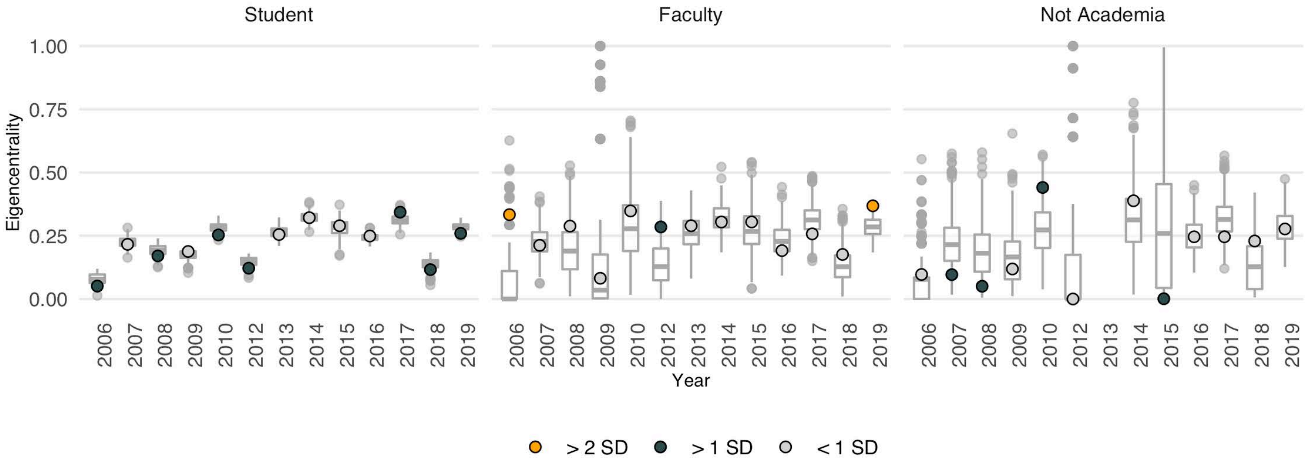

S9 Fig: Comparison of eigencentrality values aggregated by participant institutional prestige to null model. Dots correspond to actual eigencentrality value. Grey error bars represent 95th percentile confidence intervals from the null model.

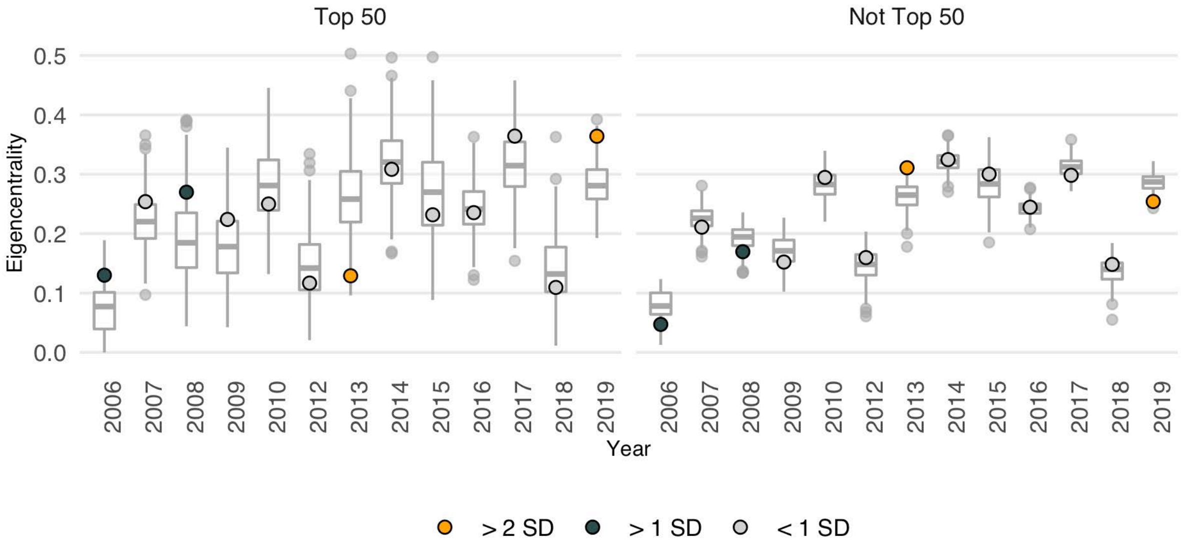

S10 Fig: Comparison of eigencentrality values aggregated by participant country of study. Dots correspond to actual eigencentrality value. Grey error bars represent 95th percentile confidence intervals from the null model.

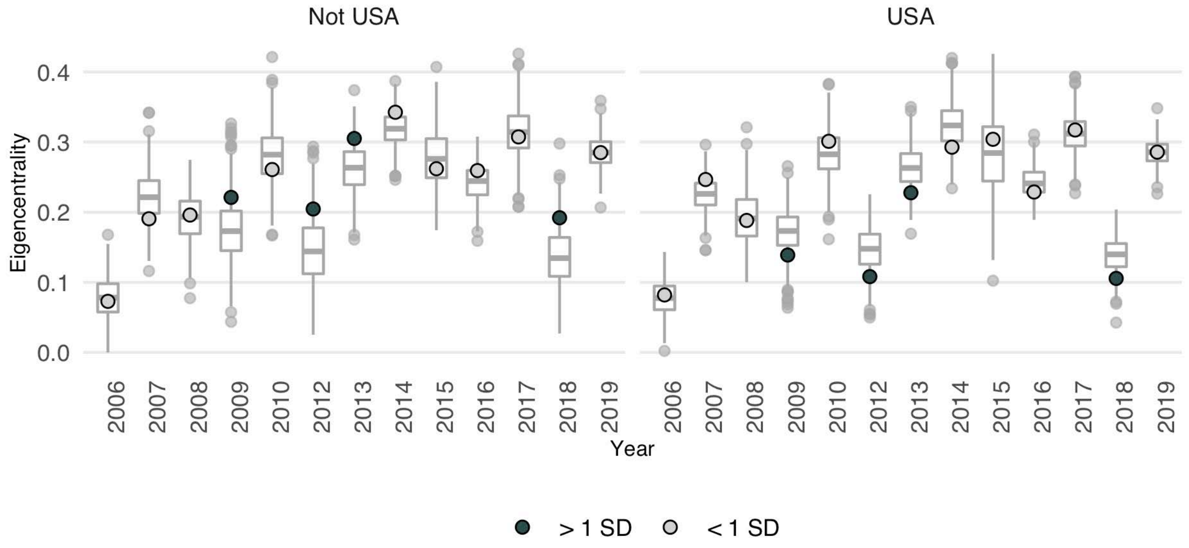

## C Project Topics and Participant Discipline Proportions by Year

S11 Fig: Comparison of the proportion of participants from each discipline to the proportion of project topics in each discipline by year. Points above the line have more projects than expected based on the number of participants; points below the line have fewer projects than expected. Labeled points had a difference in proportion greater than ±0.01.

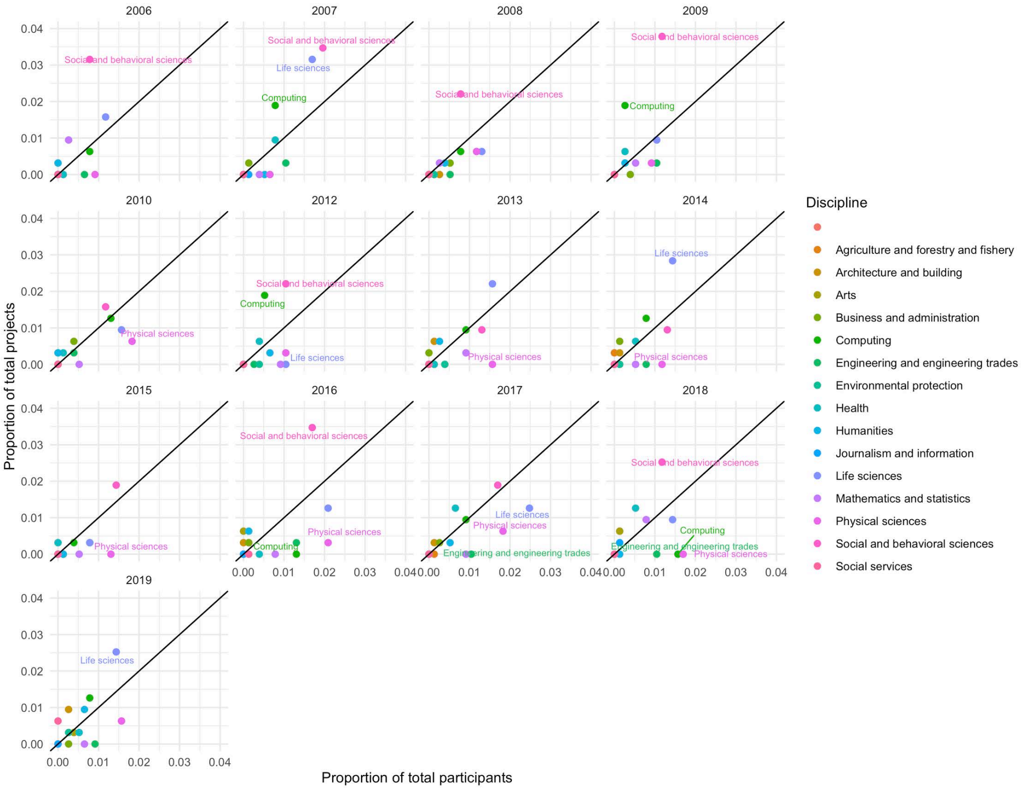

# D Collaboration Networks

S12 Fig: Collaboration Network for Santa Fe Complex Systems Summer School 2005. Nodes represent participants and links between nodes indicate collaboration between participants on a project. Both nodes and links are color-coded by academic discipline.

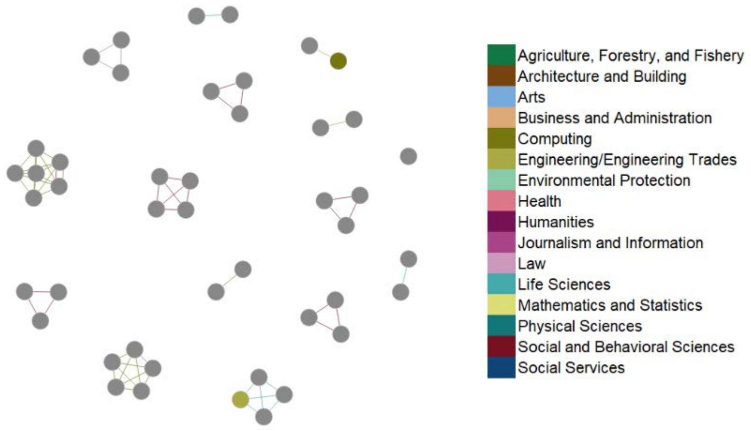

S13 Fig: Collaboration Network for Santa Fe Complex Systems Summer School 2006. Nodes represent participants and links between nodes indicate collaboration between participants on a project. Both nodes and links are color-coded by academic discipline.

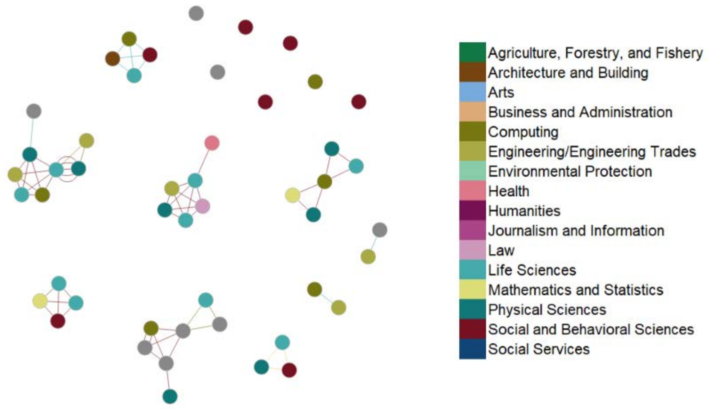

S14 Fig: Collaboration Network for Santa Fe Complex Systems Summer School 2007. Nodes represent participants and links between nodes indicate collaboration between participants on a project. Both nodes and links are color-coded by academic discipline.

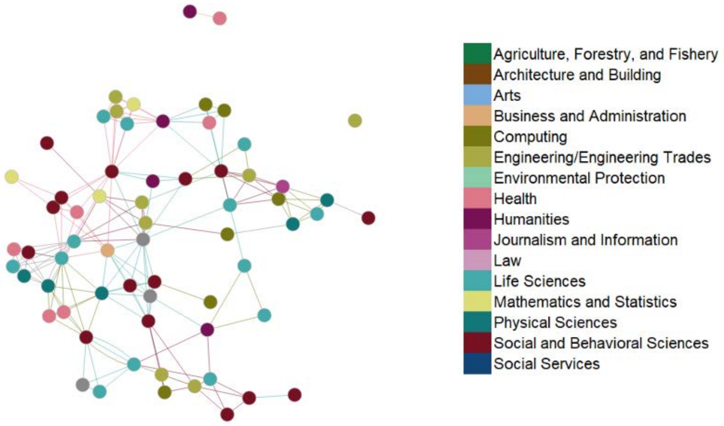

S15 Fig: Collaboration Network for Santa Fe Complex Systems Summer School 2008. Nodes represent participants and links between nodes indicate collaboration between participants on a project. Both nodes and links are color-coded by academic discipline.

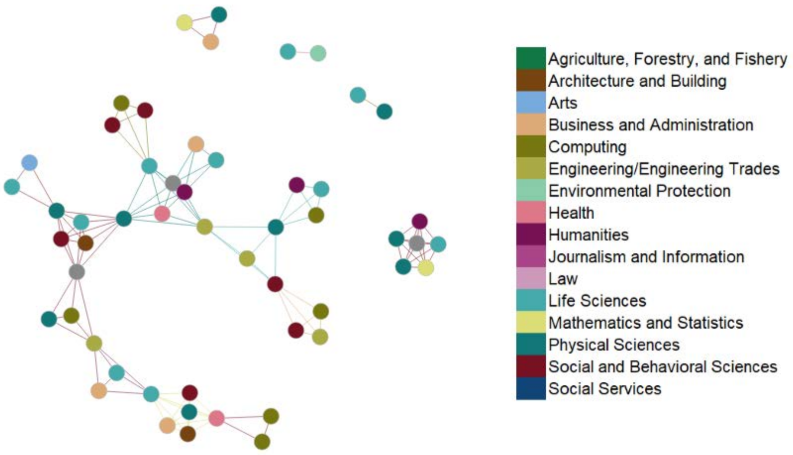

S16 Fig: Collaboration Network for Santa Fe Complex Systems Summer School 2009. Nodes represent participants and links between nodes indicate collaboration between participants on a project. Both nodes and links are color-coded by academic discipline.

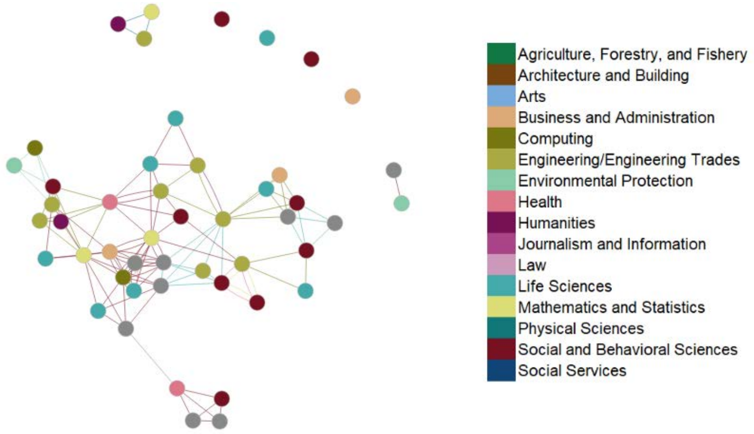

S17 Fig: Collaboration Network for Santa Fe Complex Systems Summer School 2010. Nodes represent participants and links between nodes indicate collaboration between participants on a project. Both nodes and links are color-coded by academic discipline.

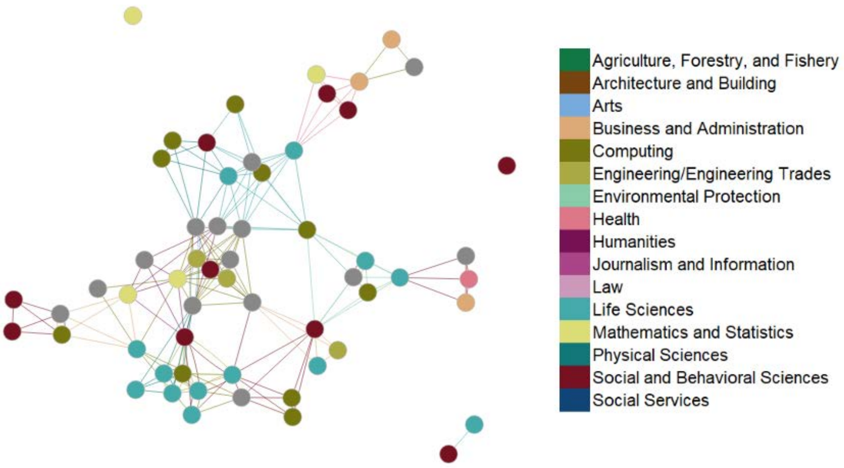

S18 Fig: Collaboration Network for Santa Fe Complex Systems Summer School 2012. Nodes represent participants and links between nodes indicate collaboration between participants on a project. Both nodes and links are color-coded by academic discipline.

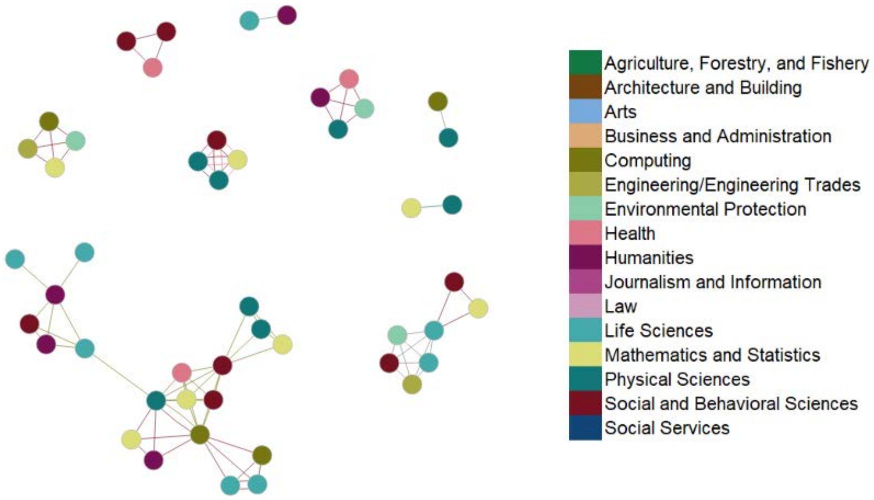

S19 Fig: Collaboration Network for Santa Fe Complex Systems Summer School 2013. Nodes represent participants and links between nodes indicate collaboration between participants on a project. Both nodes and links are color-coded by academic discipline.

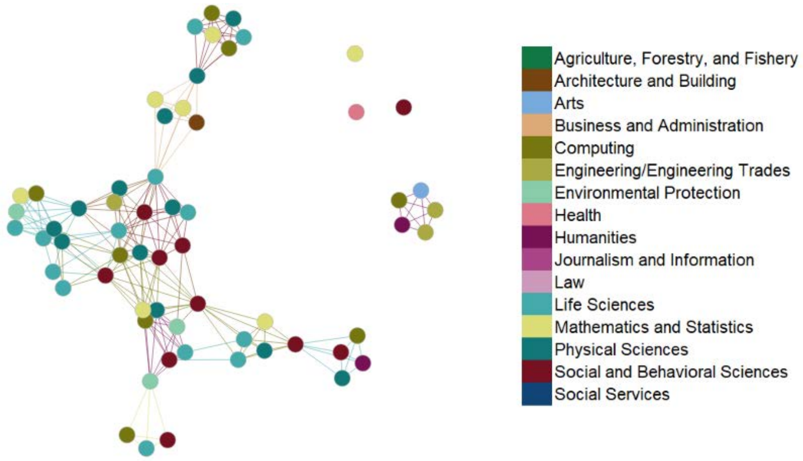

S20 Fig: Collaboration Network for Santa Fe Complex Systems Summer School 2014. Nodes represent participants and links between nodes indicate collaboration between participants on a project. Both nodes and links are color-coded by academic discipline.

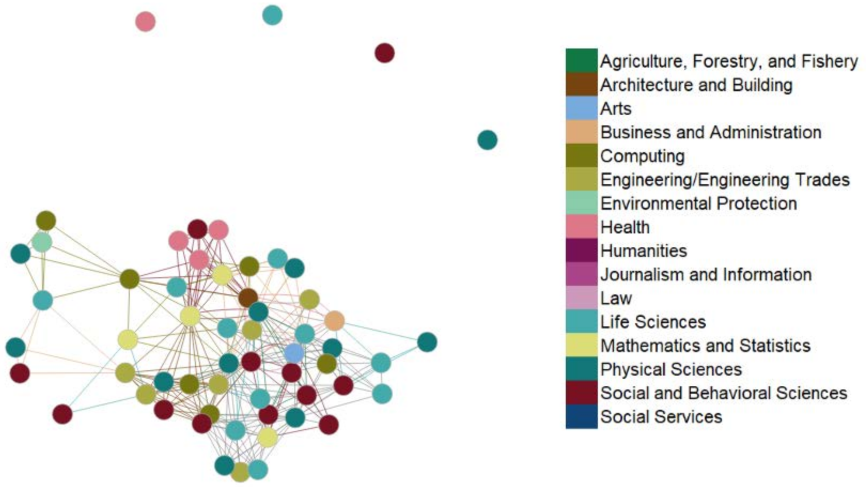

S21 Fig: Collaboration Network for Santa Fe Complex Systems Summer School 2015. Nodes represent participants and links between nodes indicate collaboration between participants on a project. Both nodes and links are color-coded by academic discipline.

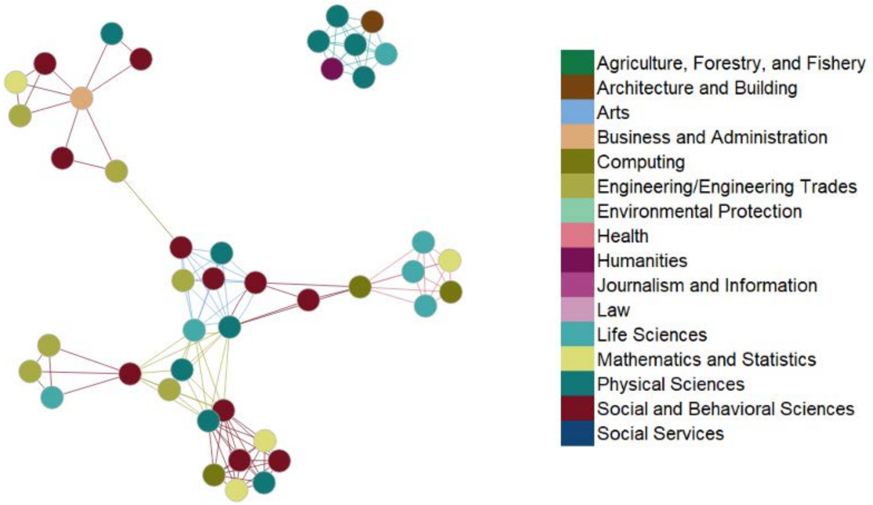

S22 Fig: Collaboration Network for Santa Fe Complex Systems Summer School 2016. Nodes represent participants and links between nodes indicate collaboration between participants on a project. Both nodes and links are color-coded by academic discipline.

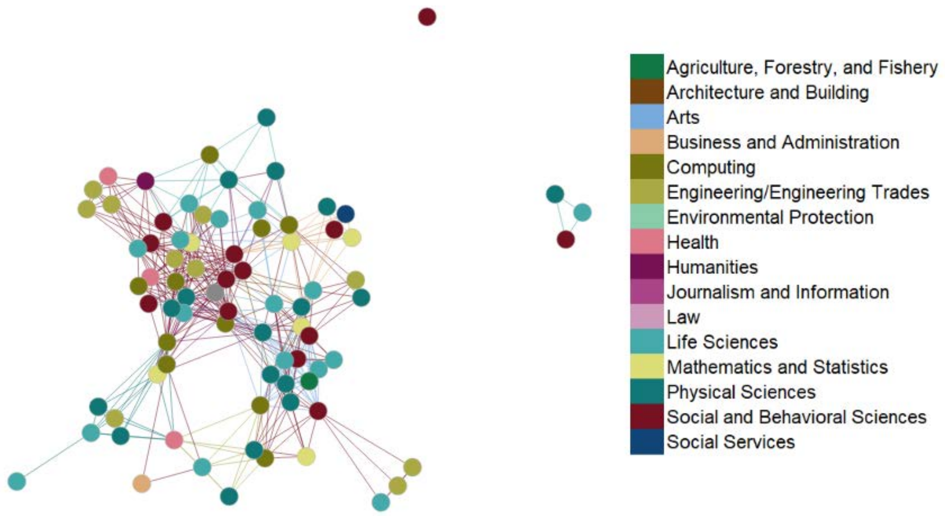

S23 Fig: Collaboration Network for Santa Fe Complex Systems Summer School 2017. Nodes represent participants and links between nodes indicate collaboration between participants on a project. Both nodes and links are color-coded by academic discipline.

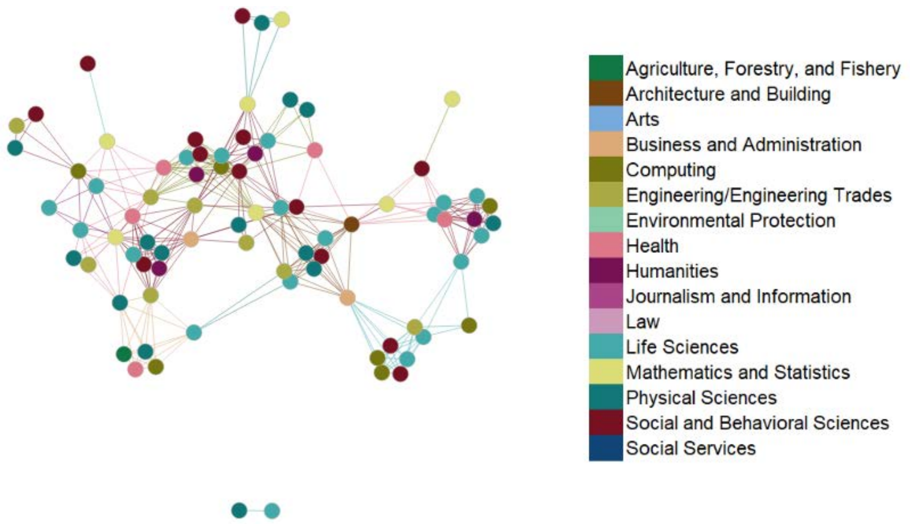

S24 Fig: Collaboration Network for Santa Fe Complex Systems Summer School 2018. Nodes represent participants and links between nodes indicate collaboration between participants on a project. Both nodes and links are color-coded by academic discipline.

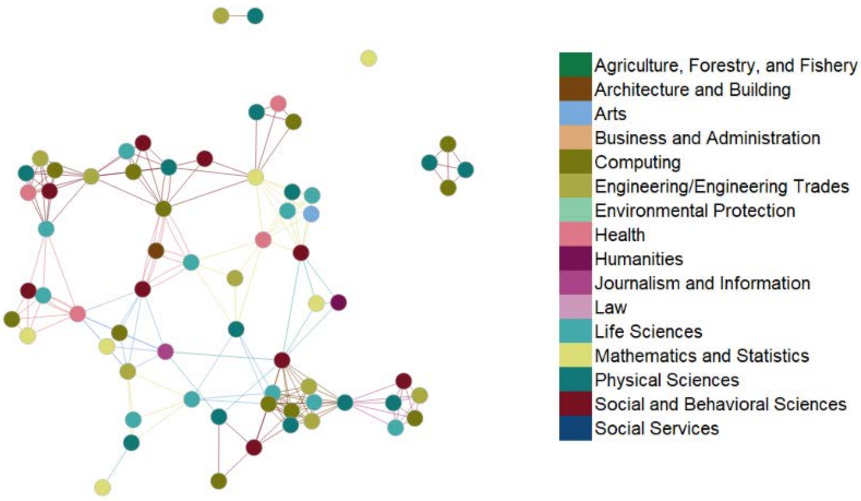

S25 Fig: Collaboration Network for Santa Fe Complex Systems Summer School 2019. Nodes represent participants and links between nodes indicate collaboration between participants on a project. Both nodes and links are color-coded by academic discipline.

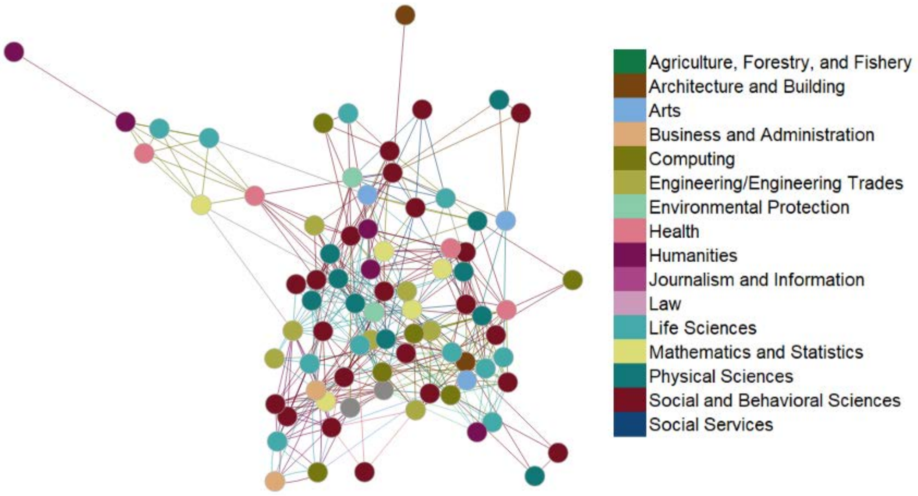

Supplement: S1 Appendix — (PDF) [file pone.0246260.s001.pdf]
