# Supplementary material for: A breeding pool of ideas: Analyzing interdisciplinary collaborations at the Complex Systems Summer School
Source: PLoS One. 2021 Feb 1;16(2):e0246260. doi: 10.1371/journal.pone.0246260 (PMC7850475; doi:10.1371/journal.pone.0246260)
Supplement: Attachment — Submitted filename: Response to Reviewers.docx [file pone.0246260.s003.docx]

January 11, 2021

Dear Dr. Lozano and Reviewers,

Thank you for your feedback. We have incorporated your comments into the most recent draft of our manuscript. Please find our response to each point written in blue text below:

1. You are using the Herfindahl Hirschman Index (HHI) as a measure of homophily. Could you, please, elaborate on the convenience of using this index instead of, for instance, assortativity?

We chose to use HHI instead of an index such as assortativity because it allows us to examine the diversity of a participant’s collaborations without considering the attributes of that particular individual. Given that not all groups are equally represented in our dataset, using a metric based on similarity between ego and the nodes in its network could potentially bias our measures of homophily of collaborations. For example, for an individual in a rare discipline like Social Services, a similarity-based homophily measure would necessarily indicate a more heterogeneous set of collaborations simply because there are no other Social Services participants to work with. It is possible, however, that Social Services participants exclusively work with individuals from the Social and Behavioral Sciences, meaning that their collaboration networks are actually not diverse. Using HHI allows us to see this information. Later on in the paper, we use a dyad analysis to examine the tendency of individuals to work with others from their discipline; if we had used a similarity-based homophily measure, we would be doubly analyzing this phenomenon. We have added language summarizing these points in Section 2.3:

*“HHI measures the concentration of a node's ego network in particular groups without considering the node's own attributes. This allows for more clearly assessing the diversity of a node's collaboration network without requiring the in-group and out-group dichotomy based on similarity involved in many other homophily measures. By using HHI, we can more directly address the question of whether participants are working with a diverse network of people, as opposed to whether they are collaborating with individuals similar to themselves. The latter is investigated via a dyadic analysis, which is explained below.”*

2. The boxplot in Fig3 shows that some disciplines (e.g. Social and behavioural sciences) present a significant amount of values equal to one (so, all groups composed by a single discipline). Have you checked whether this is related to smaller group sizes? As you normalized 'discipline diversity' by group size (to be able to compare across cases) this cannot be checked directly.

This is a good question. We looked into it and found that for all disciplines in which completely homogenous groups occur, the median and mode size of these groups is 2. However, we still found an overall pattern of diversity in group composition, which we tested first by excluding groups of 2 from the data, and then by considering only groups that fall within the interquartile range (3 to 6 individuals, inclusively). We have revised Section 3.2 accordingly:

*“This metric is somewhat sensitive to group size, considering that smaller groups are more likely to have extreme values (either completely homogeneous or completely heterogeneous group composition). Fields where the modal group size is only two, such as  Life Sciences and Computing, may be especially sensitive (see Table~\ref{tab:Disc-Table}). Despite this group size effect, there is an overall pattern of high diversity, which remains when only particular subsets of the data are considered, such as all groups comprising more than 2 people or group sizes that fall within the interquartile range (3 to 6 people, inclusively).”*

We have also updated the caption of Fig3 to add clarity:

*“Proportion of unique disciplines of participants within a project group averaged over all years of the Complex Systems Summer School summarized by topic of the project. Values of 1 indicate that every member of a group is from a different discipline. Values of 0 mean that all group members are from the same discipline.”*

In addition, we have updated Table 2 to include the mean, median, and modal group size for each discipline.

3. In page 7, first paragraph, the text reads "Specifically, disciplinary HHI was above one standard deviation above the expected for 2012, 2013, 2016, and 2018." Maybe this sentence could be re-written to avoid using 'above' twice.

We have re-written this sentence to say the following:

*“Specifically, disciplinary HHI was greater than one standard deviation above the expected for 2012, 2013, 2016, and 2018.”*

Finally, we have made minor tweaks to the manuscript, as well as identifying and fixing an error in our data processing, leading to 823 rather than 824 participants, and 322 rather than 321 projects. We have also updated the figures that relied on this data.

We hope that we have addressed each point satisfactorily.

Sincerely,

Jacqueline Brown, Dakota Murray, Kyle Furlong, Emily Coco, and Fabian Dablander
